# Procedural and Short-Term Outcomes of Carotid Artery Stenting: A Single-Center Experience

**DOI:** 10.7759/cureus.75763

**Published:** 2024-12-15

**Authors:** Sitaram Barath, Ramesh Patel, Gaurav Kumar Mittal, Rishabh Mohindru, Jai Bharat Sharma, Dilip Jain

**Affiliations:** 1 Department of Radiology, Geetanjali Medical College and Hospital, Udaipur, IND; 2 Department of Cardiology, Geetanjali Medical College and Hospital, Udaipur, IND

**Keywords:** carotid artery, embolic protection device, ischemic stroke, self-expanding carotid stent, stenting

## Abstract

Background

Carotid artery stenting is a well-established alternative treatment to carotid endarterectomy for carotid artery stenosis for preventing stroke. This study assessed the procedural and clinical outcomes in patients undergoing carotid artery stenting in a tertiary care center in India.

Methods

A total of 39 patients underwent carotid artery stenting from January 2022 to December 2023, with different embolic protection devices and carotid stents. All the patients had symptomatic carotid artery stenosis with at least 50% stenosis as per the North American Symptomatic Carotid Endarterectomy Trial (NASCET) criteria. Procedural and technical success was assessed, and patients were followed for 20 ± 7 months for survival, new-onset stroke, and quality of life.

Results

The mean age was 64.8 ± 9.1 years and 25 (64.1%) of the patients were males. A total of 26 (66.7%) patients were hypertensive and 21 (53.9%) patients had a history of diabetes mellitus. Out of 39 patients, 2 (5.1%) had recurrent transient ischemic attacks while the rest (37; 94.9%) had a subacute and chronic ischemic stroke. A total of 37 (94.9%) patients had carotid artery stenosis of more than 70%. In this study, 9 (23.1%) patients had a history of sub-acute ischemic stroke of a 1 to 3-week duration. Two patients underwent bilateral staged stenting over a gap of one month. The overall procedural success rate was 39 (100%) and none of the patients had access site-related major complications. Out of the total 41 implants, 21 (51.2%) were self-expanding carotid WALLSTENT (Boston Scientific, Marlborough, Massachusetts, US) and 20 (48.8%) implants were Protégé RX tapered self-expanding carotid stent (Medtronic, Dublin, Ireland). A FilterWire EZ (Boston Scientific) embolic protection device was used in 23 (56.1%) cases and Spider FX (Medtronic) in 18 (43.9%) cases. None of our patients had an intraprocedural death. One patient had postprocedural ipsilateral third nerve palsy, which was resolved partially on the next day of carotid angioplasty. We used dual-antiplatelet drug therapy post-procedure for a month followed by a single antiplatelet drug lifelong. All patients were followed for a minimum of six months and a maximum of 30 months. A total of three (7.7%) patients have died, and 2 (5.1%) patients had a new-onset ischemic stroke with one on the same side of the carotid stenting. A total of 31 (79.5%) patients were doing well and in the recovery phase while those two patients who had a recurrent stroke were bedridden. None of the patients had clinically significant restenosis that needed revascularization of the carotid artery over a mean follow-up period.

Conclusion

Carotid artery stenting is an effective method to reduce the recurrence of ischemic stroke in significant symptomatic carotid stenosis patients. Experience in neuro-interventional procedures at our center leads to an acceptable rate of peri-procedural stroke, recurrence, and mortality in carotid artery stenting procedures.

## Introduction

Stroke is one of the leading causes of adult disability and mortality, and most of them are ischemic stroke. Nearly 20% of ischemic strokes are from atherosclerotic disease that affects extra and intracranial arterial circulation and thus remains an important target for stroke prevention [1]. Asymptomatic carotid stenosis >70% is associated with a 1.5-2% yearly risk of ipsilateral stroke while >90% disease is associated with a stroke risk of 2.5% yearly [2-5]. Symptomatic carotid stenosis patients had a higher risk of stroke, in these, with stenosis of 70-89%, the yearly risk of ipsilateral stroke is ~10% while the risk increases to 35% in those with stenosis of 90-95%, but is lower, at 10%, in those with symptomatic subtotal carotid occlusion while complete occlusion is usually asymptomatic and is associated with a rather low stroke risk <1% [6-8]. Revascularization by carotid endarterectomy or using carotid stenting is a well-known method to reduce the risk of stroke in carotid artery disease patients. We report procedural methods and follow-up data in our carotid stenting patients.

## Materials and methods

Study design and population

This was an observational study with a bidirectional cohort of 39 patients who underwent carotid artery stenting from January 2022 to December 2023 at a tertiary care center in India.

Data collection and methodology

Institutional ethics committee approval was taken. Patients were identified from electronic medical records. Informed consent was taken. We followed the 2011 American Stroke Association guidelines for the cut-off standard at our center [9]. The minimum gap in acute stroke patients from the day of the last stroke was a minimum of one to two weeks depending on infarct size. At our center, we did not intervene for thrombotic occlusion and chronic total occlusion in carotid arteries.

Endpoints and follow-up

Our outcome variables include new onset stroke and death. The patients were followed in person at the outdoor department or by telephone for a minimum of six months. The maximum follow-up duration in our study was 30 months. Three questionnaires were scrutinized for each participant; “Is the patient alive or died, if death occurred possible cause of death was scrutinized”; “If alive any recurrent stroke”; and lastly, “Did the quality of life improve after the index procedure?”.

Procedural methods

The magnetic resonance imaging stroke protocol with magnetic resonance angiography, including carotid and vertebral angiogram, was a usual protocol for stroke evaluation in our institute. We did not mandate carotid Doppler for the preprocedural evaluation but used it for follow-up in-stent restenosis evaluation. The pre‐procedural assessment includes a meticulous history of comorbidities, neurological examination, and calculation of the National Institutes of Health Stroke (NIHSS) scale. Routine blood investigations, including complete blood count, and renal profile were evaluated in every patient. We followed North American Symptomatic Carotid Endarterectomy Trial (NASCET) methods to define treatment cut-offs that measured stenosis in reference to distal internal carotid artery diameter and gave a more conservative estimate than the European Carotid Surgery Trial (ECST) method [6,8].

The procedure was performed under local anesthesia unless there was a specific need for general anesthesia. The trans-femoral approach was our first choice. Access was obtained with a 5-Fr femoral sheath using the Seldinger technique. The intraprocedural target for activated clotting time was 200-250 seconds. A 5‐Fr Judkins right or Picard angiographic catheter was initially used for arch aortogram and a selective carotid angiogram for road mapping. A 7‐Fr, 90‐cm Flexor Shuttle sheath (Cook Medical, Bloomington, Indiana, US) was exchanged using a 0.035‐inch J tip hydrophilic guidewire (Terumo, Shibuya, Tokyo, Japan) and advanced into the common carotid artery below the bifurcation with gentle manipulation. Before cannulation of the common carotid, careful aspiration and flush were performed. We use the ‘no‐touch technique’ to minimize the risk of embolism or dissection of the common carotid artery [10]. An angiogram of the carotid with intracerebral vessels was taken using a minimum of two projections, we commonly take angiograms in lateral view and anteroposterior 30° cranial.

We parked an embolic protection device in the carotid artery with a minimum of 3 cm distal to the lesion over a 0.14” filter wire. We prefer to predilate lesions with a 2 to 3-mm coronary angioplasty balloon at nominal pressure except in thrombotic lesions. We did not use atropine for prophylaxis pre-dilation, but we used it for treatment. We usually do not lower the blood pressure beforehand in hypertensives as balloon dilation causes hypotension by the vagal response. The stent diameter was based on the proximal landing zone, which should be 1-2 mm larger than the common carotid artery. The length of the stent was based on the lesion length, as the stent should cover the lesion fully with a minimum of 5 mm normal-appearing landing zone proximally and distally. After stent deployment, we routinely did not do post-balloon dilation and performed only in required cases with a coronary balloon equal to the distal internal carotid artery diameter except in a thrombotic lesion. Final carotid angiogram, including intracranial branches to document the final result and to exclude distal embolization. Filter device removal with a retrieval catheter was done carefully. Continuous monitoring of the heart rate, blood pressure, and neurological status throughout the procedure is mandatory. Good hydration and maintenance of appropriate blood pressure (systolic blood pressure below 140 mmHg) are important during recovery. Dual antiplatelet therapy of aspirin 150 mg and clopidogrel 75 mg per day was started on the day before the elective procedure and continued for one month unless contraindicated followed by single antiplatelet therapy.

Statistical analysis

Data analysis was done using SPSS software (version 19, IBM Corp, Armonk, NY, US). Continuous variables were described as mean and standard deviations and categorical variables were described as frequency counts and percentages. P value <0.05 was considered statistically significant.

## Results

A total of 39 patients were included in the study. The mean age of the patients was 64.8 ± 9.1 years, and 25 (64.1%) patients were males. Hypertension was reported in 26 (66.7 %) patients, diabetes mellitus in 21 (53.9%) patients, and 4 (10.3%) patients had kidney injury either acute or chronic. All patients had symptomatic carotid artery stenosis of at least 50% while none of the patients had total occlusion. A total of 37 (94.8%) patients had stenosis of more than 70% by NASCET criteria while 7 (17.9%) patients had bilateral carotid stenosis of more than 50% and 3 (7.7%) patients had a history of coronary artery revascularization in the past, out of which one had undergone coronary artery bypass grafting. Out of 39 patients, 2 (5.2%) patients had a recurrent transient ischemic attack while the remaining 37 (94.9%) patients had a history of ischemic stroke. We routinely did not intervene during the first week of ischemic stroke and preferred a gap of two weeks in the case of a large infarct. In our study, 9 (23.1%) patients had a sub-acute ischemic stroke of 1- to 3-week-old while 28 (71.8%) had a chronic ischemic stroke. Baseline characteristics are shown in Table 1.

**Table 1:** Baseline characteristics CABG: coronary artery bypass grafting; TIA: transient ischemic attack

Variables	n (%)
Male	25 (64.1%)
Female	14 (35.9%)
Hypertensive	26 (66.7%)
Diabetics	21 (53.9%)
Renal injury (acute or chronic)	4 (10.3%)
History of coronary artery disease	3 (7.7%)
Post CABG	1 (2.5%)
Diagnosis	
Recurrent TIA	2 (5.2%)
Subacute Ischemic stroke	9 (23.1%)
Chronic ischemic stroke	28 (71.8%)
Stenosis	
50-70%	2 (5.2%)
70-95%	32 (82%)
95-99%	5 (12.8%)
100%	0
Bilateral	7 (17.9%)

All the cases proceeded with the femoral route. Two patients underwent bilateral staged carotid stenting over a gap of a minimum of one month. Out of 41 stents that were implanted in 39 patients, 21 (51.2%) were implanted with self-expanding carotid WALLSTENT (Boston Scientific) and 20 (48.8%) implants were Protégé RX tapered self-expanding carotid stent (Medtronic). We used the embolic protection device in all patients, with FilterWire EZ (Boston Scientific) protection in 23 (56.1%) cases and Spider FX (Medtronic) in 18 (43.9%) cases. None of the patients had access site-related major complications. None of our patients had an intraprocedural death. We routinely take post-procedural cerebral artery angiogram post-stenting to rule out intraprocedural embolization. In our study, one patient had postprocedural ipsilateral third nerve palsy causing ipsilateral ptosis, squint, and diplopia, which resolved partially on the next day and managed conservatively.

**Figure 1:**
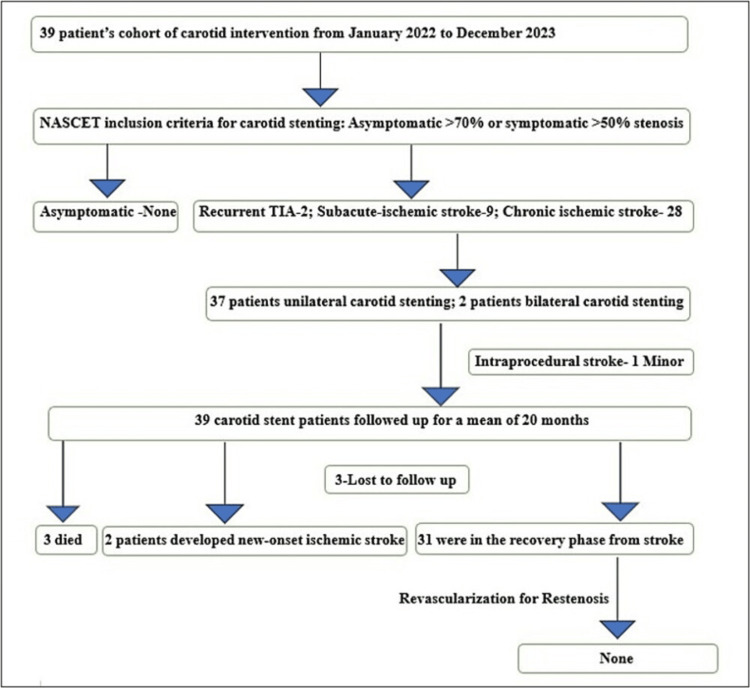
Study flow chart NASCET: North American Symptomatic Carotid Endarterectomy Trial; TIA: transient ischemic attack. Image Credit: Dr. Gaurav Kumar Mittal

The mean follow-up duration in our study was 20 ± 7 months with a minimum follow-up of six months and a maximum of 30 months. A total of 3 (7.7%) patients were lost to follow-up and 2 (5.1%) patients had a new-onset stroke, and both were having an ischemic stroke, one was on the same side after one month of the procedure while another was on the opposite side of the carotid stent after one year of stenting and there was no complaint regarding the prescribed medication. None of the patients had developed hemorrhagic stroke or hemorrhagic conversion of ischemic stroke. A total of 3 (7.7%) patients died over a 20-month follow-up period, one due to interstitial lung disease-related complication, one due to renal dysfunction, and one had sudden cardiac death. A total of 31 (87.2%) patients were doing well and in the recovery phase while those two patients who had a recurrent stroke were bedridden. None of the patients had clinically significant Doppler-detected restenosis, which needed revascularization over the follow-up period of a mean of 20 months (Figure 1).

## Discussion

This bidirectional cohort study on 39 patients who underwent carotid artery stenting reports a mean age of 64.8 ± 9.1 years at the time of stenting, with a predominantly male population. More than half of the patients were hypertensive and diabetics, which points out the importance of medical management of these comorbidities in carotid stenting patients.

According to the 2011 American Stroke Association guidelines for symptomatic carotid artery stenosis, stenosis of >50% is a class I indication for stenting or carotid endarterectomy (CEA) while in asymptomatic patients, with carotid stenosis of >70%, CEA is a class IIa recommendation and stenting is a IIb indication [9]. In our study, all the patients had symptomatic carotid stenosis of more than 50% by an angiographic method for which they were treated.

In this study, we account that none of the patients encountered periprocedural hemorrhagic conversion of ischemic stroke. At our center, the usual strategy was to wait for at least one week in case of minor ischemic stroke and two weeks for large ischemic infarct before stenting. Careful selection of patients is important to prevent this dreaded complication.

The risk of perioperative stroke during carotid stenting is 1-2% when performed for asymptomatic carotid stenosis and 3-4% when performed for symptomatic carotid stenosis [10-12]. In our study, it was 2.5%, which was acceptable. The embolic protection devices we used in our patients were FilterWire EZ (Boston Scientific) and Spider FX (Medtronic). In this study, the failure rate of FilterWire EZ (Boston Scientific) was 2 (4.3%). The Spider RX (Medtronic) consists of a windsock‐type filter basket made of a nitinol wire mesh that has a pore size of 70-200 microns and is available in 3 to 7-mm sizes. FilterWire EZ (Boston Scientific) is mounted on a 0.014‐inch wire using an eccentric nitinol wire loop with a polyurethane membrane filter that has pores of 110 μm. An in vitro study showed a capture efficiency of 78.1% for Spider RX (Medtronic) and FilterWire EZ (Boston Scientific) was 98.1% [13-16]. Embolic protection device characteristics are shown in Table 2.

**Table 2:** Embolic protection devices

Embolic protection device	Filter material	Pore size (µm)/ Basket length (mm)	Capture wire (cm)	Device size (mm)
Spider RX	Nitinol mesh	70-200/15	190/320	3,4,5,6,7
Filter Wire EZ	Polyurethane membrane	110/10	190/300	3.5-5.5 (one size)

Our institute's usual protocol for coronary artery bypass grafting patients was to do a carotid Doppler during a preop workup. If significant stenosis is detected, we proceed with CEA unless the patient prefers stenting. Decisions to perform CEA vs. carotid stenting were individualized based on the patient’s age (>80 years), string sign and difficult anatomy (favors CEA), cardiopulmonary or neck comorbidities (favor stenting), and patient’s preference with keeping in mind the higher risk of minor stroke with stenting and the higher risk of myocardial infarction with CEA [11-14].

For patients who require bilateral stenting, we usually keep a gap of four to six weeks for staged stenting. In this study, 2 (5.1%) of patients underwent bilateral carotid stenting. The carotid stent we used for our patients was either Carotid WALLSTENT (Boston Scientific) or Protégé RX tapered carotid stent (Medtronic). Both are self-expanding stents with a 0.014’’ guidewire compatible rapid‐exchange monorail delivery system [15,16]. Carotid WALLSTENT (Boston Scientific) is a closed cell design stent made up of Conichrome and comes in a diameter of 6-10 mm and a length of 22-37 mm when fully opened. A disadvantage is that the stent straightens the vessel more than open‐cell nitinol stents. Protégé Carotid Stent (Medtronic) is an open-cell design stent made of nitinol with a straight or tapered design. The straight stent has a diameter of 6-10 mm and a length of 20-60 mm and the tapered one has a diameter of 8-6 mm or 10-7 mm and a length of 30 or 40 mm. The stent does not shorten during implantation. Carotid stent sizing is represented in Table 3.

**Table 3:** Carotid stents

Carotid Wallstent			Protégé RX tapered Stent	
Unconstrained Diameter (mm)	Vessel Diameter (mm)	Implanted Length (mm)	Stent Diameter and length (mm)	Reference vessel Diameter (mm)
6	5	30	8-6 × 30 or 40 mm	6.5-7.5 - (4.5-5.5)
8	7	30		
8	7	40		
8	7	50	10-7 × 30 or 40 mm	8.5-9.5 - (5.5-6.5)
10	9	30		
10	9	40		
10	9	50		

Pre‐dilatation is done to facilitate the introduction of the stent delivery system in cases of very tight or calcified carotid artery stenosis. Post-stent dilatation of the stent segment in the common carotid artery is not usually necessary. We recommend at least 5 mm extra landing from the lesion site proximally and distally [17]. The oversizing of a stent in the internal carotid artery does not cause problems. Covering the external carotid artery (ECA) is safe and rarely causes occlusion of the ECA and even if the ECA becomes significantly stenosed or occluded, this does not cause symptoms and does not need treatment.

The recurrent stroke rate in this study was 2 (5.1%), which was partially related to non-compliance with medication. The mortality rate over a 20-month follow-up period was 3 (7.7%), and all deaths were non-stroke-related causes. A total of 31 (79.5%) of the patients were doing well and in the recovery phase while those two patients who had a recurrent stroke were bedridden.

In our study, none of the patients developed Doppler measured clinically significant restenosis rate, which needed revascularization after a mean follow-up of 20 months. There is a risk of neointimal hyperplasia early on, and recurrent atherosclerosis later causing a restenosis risk of 5-10%, mainly in the first 18 months [18].

The current medical treatment of carotid artery stenosis includes smoking cessation, inhibition of platelets by antiplatelets, lowering cholesterol levels with statin therapy, and pharmacological treatment of hypertension and diabetes, if present. We use a combination of aspirin 150 mg and clopidogrel 75 mg for a month followed by aspirin monotherapy. A two-week duration of dual antiplatelet therapy may be acceptable if necessary [19,20].

Study limitations

Our study had several limitations, including its retrospective nature with a limited number of patients and the shorter duration of follow-up. These results further need to be studied with a larger sample size and long-term follow-up.

## Conclusions

Carotid stenting is a safe and effective method to prevent ischemic stroke. While compliance with antiplatelets is important to gain a better success rate, the duration of dual antiplatelet therapy can be debated. Experience in other neuro-interventional procedures generates additional excellence that is required for delegation of the carotid artery stenting procedure and dealing with complications.
